# 785. Bacterial Co-infection and Empirical Antibiotic Therapy in Patients with COVID-19

**DOI:** 10.1093/ofid/ofac492.046

**Published:** 2022-12-15

**Authors:** Jiyoung Lee, Jiwon Jung, Min Jae Kim, Yong Pil Chong, Sung-Han Kim, Sang-Oh Lee, Sang-Ho Choi, Yang Soo Kim, SeongMan Bae

**Affiliations:** Asan medical center, Seoul, Seoul-t'ukpyolsi, Republic of Korea; Asan Medical Center, Seoul, Seoul-t'ukpyolsi, Republic of Korea; Asan Medical Center, Seoul, Seoul-t'ukpyolsi, Republic of Korea; Asan Medical Center, Seoul, Seoul-t'ukpyolsi, Republic of Korea; Asan medical center, Seoul, Seoul-t'ukpyolsi, Republic of Korea; Asan Medical Center, Seoul, Seoul-t'ukpyolsi, Republic of Korea; Asan Medical Center, Seoul, Seoul-t'ukpyolsi, Republic of Korea; Asan Medical Center, Seoul, Seoul-t'ukpyolsi, Republic of Korea; Asan medical center, Seoul, Seoul-t'ukpyolsi, Republic of Korea

## Abstract

**Background:**

Understanding the rate and composition of bacterial co-infection is important to determine antibiotic therapy in SARS-CoV-2 infection, but those vary according to healthcare settings and regional differences. We evaluated the rate of bacterial co-infection in hospitalized patients with COVID-19 in a single tertiary hospital in South Korea.

**Methods:**

In this retrospective study, all the adult patients with COVID-19 hospitalized between Feb 2020 and Dec 2021 were included. Bacterial co-infection rate was assessed by results of sputum cultures, blood cultures, pneumococcal urinary antigen, Legionella urinary antigen, sputum *Legionella pneumophilia* PCR, and sputum multiplex PCR for *Mycoplasma pneumoniae* and *Chlamydia pneumoniae*. Characteristics and outcomes of patients were evaluated according to antibiotics exposure prior to hospitalization.

**Results:**

Of 367 adult patients, 300 (81.7%) patients having sputum culture results were included in the analysis. Of these, 127 (42.3%) had a history of antibiotic exposure within 1 month before hospitalization. The coinfection rate within 48 hours of hospitalization was confirmed in 8.3% (25/300): 6.4% (11/163) of patients without prior antibiotic exposure and 11% (14/127) of patients with prior antibiotic exposure. In the group without prior antibiotic exposure, pathogens responsible for community-onset infections were isolated, whereas nosocomial pathogens were predominantly isolated in the antibiotic-exposed group. Empirical antibiotics were used in 144 (66%) of 275 patients without positive results for microbiological tests. Empirical antibiotic use in patients without positive results for microbiological tests was not significantly associated with 30-day mortality or in-hospital mortality after adjusting covariates including age, sex, comorbidity, anti-inflammatory treatment, and COVID-19 severity.

**Conclusion:**

In this study with a high rate of microbiological testing, bacterial co-infection was not frequent, and the results varied depended on previous exposure to antibiotics. Given the rarity of bacterial co-infection and the lack of potential benefits of empirical antibiotic therapy, the antibiotic use in patients with COVID-19 should be restricted as an important target of antibiotic stewardship.

**Disclosures:**

**All Authors**: No reported disclosures.

